# Bioprotection of *Zea mays* L. from aflatoxigenic *Aspergillus flavus* by *Loigolactobacillus coryniformis* BCH-4

**DOI:** 10.1371/journal.pone.0271269

**Published:** 2022-08-02

**Authors:** Mahwish Salman, Muhammad Rizwan Javed, Hazrat Ali, Ghulam Mustafa, Anam Tariq, Tanzila Sahar, Shazia Naheed, Iqra Gill, Muhammad Abid, Abdul Tawab

**Affiliations:** 1 Department of Biochemistry, Government College University Faisalabad (GCUF), Faisalabad, Pakistan; 2 Department of Bioinformatics and Biotechnology, Government College University Faisalabad (GCUF), Faisalabad, Pakistan; 3 National Institute for Biotechnology and Genetic Engineering (NIBGE), Faisalabad, Pakistan; 4 Department of Biochemistry, Government College Women University Faisalabad, Faisalabad, Pakistan; 5 Department of Chemistry, Government College University Faisalabad (GCUF), Faisalabad, Pakistan; 6 Department of Statistics, Government College University Faisalabad (GCUF), Faisalabad, Pakistan; Universita degli Studi di Pisa, ITALY

## Abstract

Fungal infection causes deterioration, discoloration, and loss of nutritional values of food products. The use of lactic acid bacteria has diverse applications in agriculture to combat pathogens and to improve the nutritional values of cereal grains. The current research evaluated the potential of *Loigolactobacillus coryniformis* BCH-4 against aflatoxins producing toxigenic *Aspergillus flavus* strain. The cell free supernatant (CFS) of *Loig*. *coryniformis* was used for the protection of *Zea mays* L. treated with *A*. *flavus*. No fungal growth was observed even after seven days. The FT-IR spectrum of untreated (T1: without any treatment) and treated maize grains (T2: MRS broth + *A*. *flavus*; T3: CFS + *A*. *flavus*) showed variations in peak intensities of functional group regions of lipids, proteins, and carbohydrates. Total phenolics, flavonoid contents, and antioxidant activity of T3 were significantly improved in comparison with T1 and T2. Aflatoxins were not found in T3 while observed in T2 (AFB1 and AFB2 = 487 and 16 ng/g each). HPLC analysis of CFS showed the presence of chlorogenic acid, *p*-coumaric acid, 4-hydroxybenzoic acid, caffeic acid, sinapic acid, salicylic acid, and benzoic acid. The presence of these acids in the CFS of *Loig*. *coryniformis* cumulatively increased the antioxidant contents and activity of T3 treated maize grains. Besides, CFS of *Loig*. *coryniformis* was passed through various treatments (heat, neutral pH, proteolytic enzymes and catalase), to observe its stability. It suggested that the inhibitory potential of CFS against *A*. *flavus* was due to the presence of organic acids, proteinaceous compounds and hydrogen peroxide. Conclusively, *Loig*. *coryniformis* BCH-4 could be used as a good bioprotecting agent for *Zea mays* L. by improving its nutritional and antioxidant contents.

## Introduction

*Aspergillus* is a filamentous fungus that produces mycotoxins (secondary metabolites), the main contaminants of food and cause adverse effects on human and animal health. The consumption of these toxins may lead to immunosuppressive, mutagenic, and carcinogenic diseases [1]. Various species of the genus *Aspergillus* produce aflatoxins such as *Aspergillus flavus*, *A*. *parasiticus A*. *nomius*, *A*. *pseudotamarii*, *A*. *bombycis*, and *A*. *ochraceoroseus* [2, 3]. Among them, *A*. *flavus* is the major food spoiling filamentous aflatoxigenic fungus [4]. It contaminates varieties of crops (e.g., maize, cottonseeds, peanuts etc.) in the field while the growth and production of aflatoxins have also been detected during post-harvest storage [5–7]. Aflatoxin B1 (AFB1) and B2 (AFB2) are important in crops and agricultural commodities [1]. Consumption of aflatoxins contaminated foods and feeds is a serious problem from the viewpoint of not only public health, but also economic losses [8].

*Zea mays*. L (Maize) is the most important cereal grain, used not only as a staple food but also as animal feed, bioenergy, and industrial crop [9]. Globally, 1.11641 billion tons of maize were produced in the year 2020 and an increase by 1.57% was expected in the year 2021 [10]. It is cultivated in South America, North America, Asia, South Africa, and North Africa, showing that this crop has worldwide adaptability. It might overtake wheat as a substantially grown crop worldwide in the coming decades. It was estimated that there were 216 million (M) maize farms globally in 2020, which is estimated to increase by 5% to 227 M by 2030 [11]. But besides several other threats, various fungi, including *A*. *flavus*, negatively affect maize by lowering its nutritional values, discoloration, decreased yield and quality, aflatoxins contamination, and oxidation that cause deterioration of food quality by disrupting its flavor and odor [12–15].

Many synthetic chemicals *viz* nitrates, benzoates, sorbates [16], and synthetic antioxidants namely BHA (butylated hydroxyanisole), BHT (butylated hydroxytoluene), and PG (n-propyl gallate) have been used for the protection of maize grains. However, the use of such chemicals and antioxidants have negative effects on the nutritional contents of cereals and cause potential risk to human health. Therefore, the use of such chemicals have been restricted in many countries [17, 18]. Consequently, an eco-friendly and bioprotective attempt is preferred to explore microorganisms with probiotic status. The cereal grains, protected with such approaches, impart beneficial health properties to consumers. The consumption of such grains improves the immune response of end -users against various diseases [19, 20].

Lactic acid bacteria (LAB) such as *Lactobacillus plantarum* [21], *Lactobacillus acidophilus* [22], and *Lactobacillus fermentum* [23] were generally recognized as safe (GRAS) and used to improve the shelf life of food. LAB have a long history of consumption and applications in food processing [24]. These microbes are used as a good alternative to synthetic chemicals [25]. Therefore, LAB have been considered as promising microbes for the integration of natural bioprotective agents in food, for inhibiting fungal growth and mycotoxin production [26].

LAB produce a variety of bioactive metabolites including fatty acids, hydrogen peroxide, organic acids, bacteriocins, proteinaceous compounds, and phenolic acids *etc*. having the potential bioactivities including antifungal and antioxidant activities [21, 22, 27, 28]. Of these metabolites, the organic acids, produced by *Loig*. *coryniformis*, belong to potent antifungal class of compounds [29, 30]. It was reported earlier that the antifungal activity of individual organic acids was quite low as compared to whole *Loig*. *coryniformis* culture supernatant [31].

In the current study, *Loigolactobacillus coryniformis* BCH-4 CFS has been evaluated for bioprotection of maize grains, contaminated with toxigenic *A*. *flavus* strain. The nutritional contents of untreated (T1) and treated (T2: MRS broth + *A*. *flavus*; T3: CFS + *A*. *flavus*) maize grains were compared by using Fourier transform infrared (FT-IR) spectroscopy. Afterward, these maize grains were evaluated for the determination of total phenolic contents, total flavonoid contents, and antioxidant activity. High-performance liquid chromatography (HPLC) was performed to determine aflatoxins, present in untreated and treated maize grains. Another HPLC analysis was done to identify the presence of phenolic acids in cell free supernatant (CFS) of *Loig*. *coryniformis* BCH-4 which has been suggested to contribute in the bioprotection potential. The current study suggested the bioprotection of *Zea mays* L. against the toxicity of *A*. *flavus* along with improving the functional antioxidant contents of maize grains.

## Materials and methods

### Media, chemicals, and maize grains

MRS (De Man, Rogosa, and Sharpe) broth/agar, Vogel’s broth/agar, ethyl acetate (HPLC grade), ethanol (HPLC grade), Folin-Ciocalteu reagent, sodium carbonate, 2,2-diphenyl-1-picrylhydrazyl, aluminum chloride, sodium nitrate, sodium hydroxide, chlorogenic acid, p-coumeric acid, 4-hydroxybenzoic acid, caffeic acid, sinapic acid, salicylic acid, benzoic acid, Gallic acid, vanillic acid, kaempferol, ferulic acid, rutin, quercetin, coumarin were purchased from Sigma-Aldrich, St. Louis, MO, USA. The FMC C-7065 maize grain variety was purchased from the local grain market, Faisalabad, Pakistan.

### Microbial cultures, and growth conditions

*Loigolactobacillus coryniformis* BCH-4, previously named *Lactobacillus coryniformis* (KX388387), was isolated from fermented rice rinsed water [29]. The culture was grown in MRS medium for 48 h at 37°C and preserved as glycerol stocks at -80°C, for long term storage in 15% (v/v) glycerol. Pure culture of *A*. *flavus*, an aflatoxigenic filamentous fungus, was obtained from the Department of Bioinformatics and Biotechnology, Government College University Faisalabad, Pakistan (Accession No. MH179066) and was grown on Vogel’s agar medium in Petri plate (100 × 17 mm diameter) for 48 h at 30°C, and stored at 4°C.

### Preparation of cell-free supernatant

Six liters of *Loig*. *coryniformis* BCH-4 were cultured in MRS broth (pH 6.4 ± 0.2), in a fermenter (BioFer-010, ICCC, Islamabad, Pakistan), with constant stirring at 120 rpm, at 37°C, for 72 h. After incubation, the cell-free supernatant (CFS) was prepared by centrifugation at 6,000 rpm for 10 min, at 4°C (Z326K, Hermle, Wehingen, Germany) and then filtered through 0.22 μm pore size filters (Advantec Toyo Kaisha, Ltd., Tokyo, Japan). The prepared CFS was stored at -20°C for further use, after freeze-drying (Alpha 2–4 LSC basic, Christ, Osterode am Harz, Germany) [32].

### *In vitro* antifungal activity of *Loig*. *coryniformis* BCH-4 CFS against *A*. *flavus*

Agar well diffusion assay was performed for determining the antifungal potential of *Loig*. *coryniformis* BCH-4 CFS [31]. For this purpose, the *A*. *flavus* (10^6^ spores/mL) culture was spread over solidified Vogel’s agar medium Petri plate (100 × 17 mm diameter), using a sterile cotton swab. A 10 mg CFS was dissolved in 1 mL sterile distilled water and a total 60 μL of CFS was added in 8 mm diameter wells. Besides, a negative control was used, having a non-inoculated MRS broth medium instead of CFS. The Petri plates were evaluated for antifungal potential by measuring the zone of inhibition around the well after incubating for 48 h at 30°C.

### Effect of heat, pH, and proteolytic enzymes on antifungal activity of CFS

To determine the biochemical nature and stability of antifungal compounds, present in *Loig*. *coryniformis*, 10 mg lyophilized CFS was treated with proteinase K, pepsin, catalase, pH effect and heat toleration according to Oirdi et al. [33] with slight modifications. The sensitivity to heat treatment was investigated by subjecting the CFS to 100°C for 20 min. To evaluate the pH effect, the CFS was neutralized with 1 M NaOH solution. The enzymatic effect on antifungal activity was determined by submitting 500 μL of CFS to 20 μL of enzymes catalase (10 mg/mL, phosphate buffer; pH 7), proteinase K (10 mg/mL phosphate buffer, pH 7), and pepsin (10 mg/mL 1 M HCL, pH 2). The mixture was incubated at 30°C for 3 h and the pH of CFS was readjusted to the initial pH value before each test. In addition, MRS broth and sterile distilled water both were used as control. All the treated CFS samples were analyzed using agar well-diffusion method against *A*. *flavus* as described above.

### Bioprotection of maize grains

#### Preparation of *A*. *flavus* culture

*A*. *flavus* was grown in Vogel’s broth medium in a 250 mL sterile flask at 30°C for 7 days until sporulation occurred. The spore concentration was determined using a hemocytometer and adjusted to 10^6^ spores per mL.

#### Bioprotective effect of CFS

The bioprotective effect of *Loig*. *coryniformis* BCH-4 CFS was determined on maize grains against *A*. *flavus* as reported by Nazareth et al. [34] with slight modifications. In short, 10 g of lyophilized CFS was dissolved in 20 mL of sterile distilled water. Besides, 20 g of maize grains were soaked in CFS (500 mg CFS/g of grains) for 8 h at room temperature after washing with distilled water. After that, the CFS-treated maize grains were shifted to the Petri plate. Similarly, 20 g of maize grains were soaked in MRS broth instead of CFS to be used as control and shifted to another Petri plate. A 5 mL of *A*. *flavus* (10^6^ spores/mL) culture was spread over grains in both plates and examined for seven days, during incubation at 30°C.

### Fourier Transform Infrared Spectroscopy (FT-IR)

For the FT-IR analysis, 10 g of untreated maize grains (T1 = without any treatment) and treated maize grains (T2 = MRS broth + *A*. *flavus* (Control), and T3 = CFS + *A*. *flavus*) were milled into refined powder. The FT-IR spectrum (Tensor II, Bruker, Billerica, MA, USA) of powdered maize grain samples were recorded within the range of 4000–400 cm^−1^ absorbance mode to observe the nutritional contents of untreated and treated maize grains [35].

### Total phenolic, flavonoid contents, antioxidant activity of treated and untreated maize grains

A 10 g of untreated (T1) and treated (T2 and T3) powdered maize grain samples were dissolved in 30 mL ethyl acetate and the supernatant was collected. After extraction, the solvent was evaporated by a rotary evaporator (R-210, Buchi, Flawil, Switzerland) at 40°C. Furthermore, 1 mL extract of each treatment was dissolved in absolute ethanol [1:1 (v/v)] to be used for the determination of total phenolic and flavonoid contents, and antioxidant activity [36].

### Total phenolic contents (TPC)

Folin-Ciocalteu method was used to determine phenolic contents spectrophotometrically [37]. Briefly, 0.5 mL of 10% Folin- Ciocalteu reagent was added in the test tubes containing 1 mL extracts or treatment solution of maize grains (T1, T2, and T3). Absolute ethanol was used as blank. The aqueous solution of sodium carbonate (2.5 mL of 700 mM) was added to each reaction mixture. The reaction tubes were vortexed, covered and incubated for 2 h at room temperature. The absorbance of each reaction mixture was measured against blank at 765 nm [37]. The measurements were compared to the standard curve of Gallic acid solution, and total phenolics were expressed as milligram of Gallic acid equivalent (GAE) per mL of (T1, T2, and T3) extract (mg GAE/mL of extract).

### Total flavonoid contents (TFC)

Aluminum chloride colorimetric method was used to determine total flavonoid contents in untreated (T1) and treated extracts (T2 and T3) spectrophotometrically [38]. Briefly, 0.4 mL of 5% sodium nitrate (w/v) was added into 1 mL ethanol extracts (T1, T2, and T3) in test tubes. Absolute ethanol was used as a blank. After 5 min, 0.6 mL of 10% AlCl_3_.6H_2_O (w/v) and 1 M NaOH were added to the mixture, mixed well and absorbance was measured at 510 nm against the blank. The measurements were compared to standard curve of quercetin solution, and total flavonoid contents (TFC) were determined as mg of quercetin per mL of (T1, T2, and T3) extract (mg quercetin/mL of extract) [38].

### Antioxidant activity by DPPH assay

The antioxidant potential of treatment solutions (T1, T2 and T3) was evaluated using 2, 2-diphenyl-1-picrylhydrazyl (DPPH) scavenging assay. This method is widely used due to its simplicity, reproducibility, and stability. The method is based on reduced DPPH which, is a stable free radical, having odd electrons and it gives maximum absorbance at 517 nm (purple color) [39]. Total, 30 μL each of untreated and treated maize grain extracts were added to 1 mL of DPPH ethanol solution (0.2 mM). The reaction mixture was incubated for 30 min at room temperature. When antioxidants react with DPPH, it is reduced by capturing electrons to form DPPH-H, resulting in decolorization (yellow color) and decrease in absorbance. Besides, 1 mL DPPH ethanolic solution was used as a control and the absorbance of samples and control was determined at 517 nm [39].

Percentage inhibition (%) of extracts was calculated using the formula given below:

$$\text{DPPH}\;\text{radical}\;\text{scavenging}\;\%\text{age}=\frac{\left({\text{A}}_{0-}{\text{A}}_{1}\right)}{{\text{A}}_{0}}\times 100$$

Where, A_0_: Absorbance of the DPPH solution; A_1_: Absorbance of each maize grain extract (T1, T2, and T3).

### Aflatoxins analysis

Powdered maize grain samples (T1, T2, and T3) were added to 60 mL of methanol : water (8:2) solution and shaken for 30 min at room temperature, under dark conditions. The extracts were filtered with Whatman filter paper and 10 mL of these extracted filtrates were diluted with 70 mL of phosphate buffer saline (PBS). The samples were again filtered through a fiber-glass filter for the separation of maize pigments. Immunoaffinity columns (Aflatest, R-biopharm, France) were used for sample cleaning up before high-performance liquid chromatography (HPLC) (FINNIGAN SURVEYOR, Thermo electron Corporation, USA). The aflatoxins (AFB1, AFB2, AFG1, AFG2) were obtained from Biopure (RomerLabs, Tulln, Austria). The working serially diluted solutions of standards of AFB1, AFB2, AFG1, and AFG2 (50, 100, 200, 400, and 500 ppb) were prepared from stock solution of 0.2 ng/μL concentrations. A 25 μL each of sample and working standard solutions was passed through C_18_ HPLC column and detected at 365 nm wavelength, using fluorescence detector (SURVEYOR FL PLUS DETECTOR, Thermo Fisher, USA). Post-column online UV derivatization was performed for better aflatoxins detection. The mobile phase used was acetonitrile : water : methanol [22.5% : 55% : 22.5% (v/v)] with the flow rate of 1 mL/min [40].

### Determination of phenolic acids in L. coryniformis BCH-4

Ten grams of *Loig*. *coryniformis* BCH-4 lyophilized CFS was extracted with ethyl acetate (20 mL) in duplicate. The obtained yellowish organic layer of metabolites was concentrated by rotary evaporator (R-210, Buchi, Flawil, Switzerland) under vacuum at a temperature below 40°C [34]. The dark brown concentrated metabolites, obtained, were used for the determination of phenolic compounds in *Loig*. *coryniformis* BCH-4. The concentrate was mixed well by vortexing and passed through 0.22 μm syringe filter (Advantec Toyo Kaisha, Ltd. Tokyo, Japan), before injecting in the HPLC system (LC300, PerkinElmer, USA), using Pinnacle DB C_18_ reversed-phase column [250 mm × 4.6 mm (Internal Diameter; ID), 5 μm]. Mobile phase was consisting of 0.5% acetic acid (v/v) in double distilled water: methanol (80:20 v/v) solution with 1 min/mL flow rate, while the UV detector (Flexar UV/Vis Detector, PerkinElmer, USA) was set at 275 nm. Data was processed by Chem 3240 software, operating system, using default method for the subject channel (FXUV Det-2 1:1) [41].

### Statistical analysis

Inhibition zone was calculated as the mean ± standard deviation of three replicates (n = 3) and statistical analysis was performed by applying ANOVA, using GraphPad Prism software version 5.0 for Windows (GraphPad Software, La Jolla California, USA). Furthermore, Tukey’s test was applied to find out the significance between total phenolic, flavonoid contents, and antioxidant treatments (p ≤ 0.05), and all the data was presented as mean ± standard error.

## Results

### *In vitro* antifungal potential of *Loig*. *coryniformis* BCH-4 CFS and its stability after various treatments

**Fig 1:**
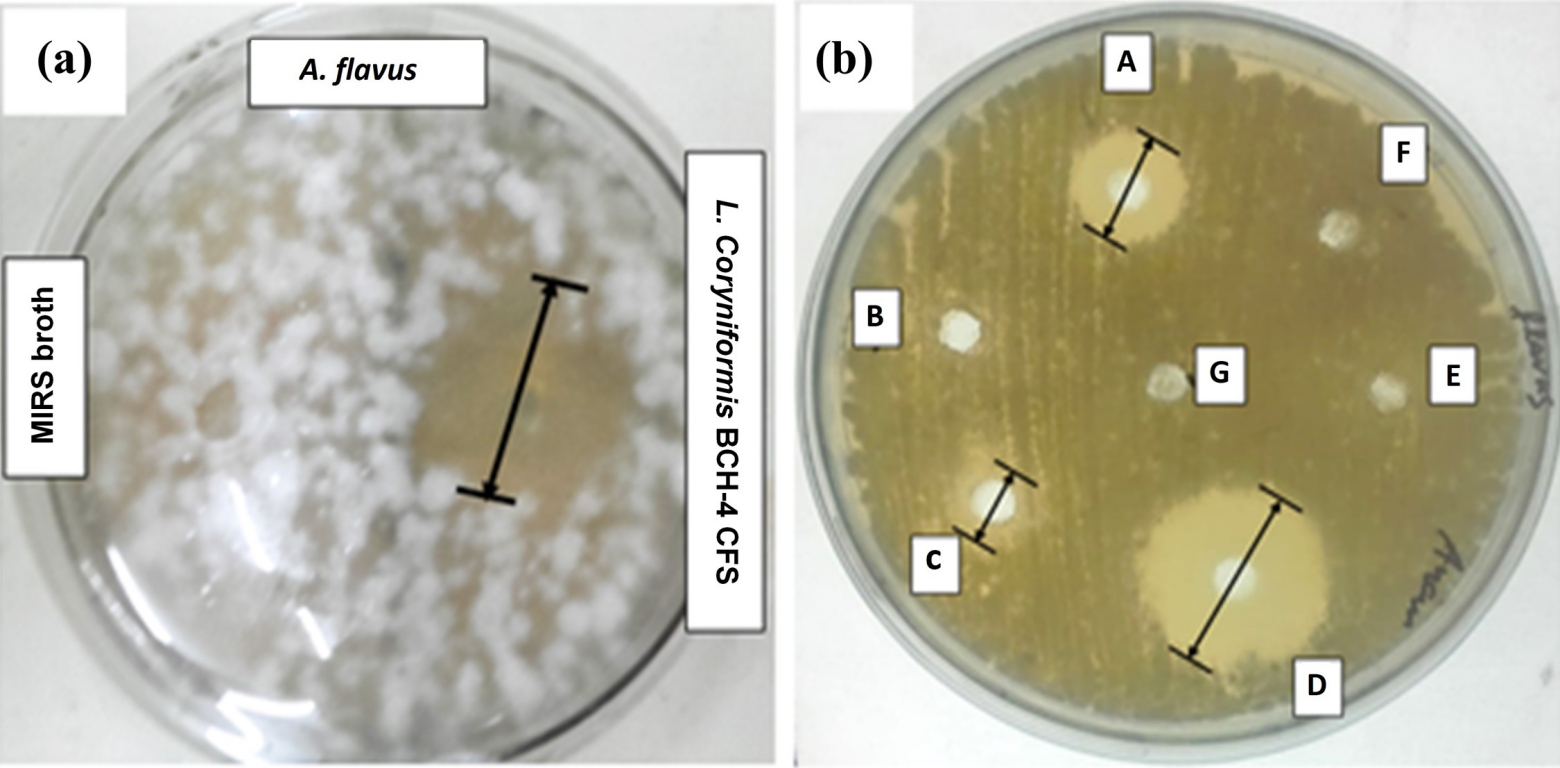
Zones of inhibition against aflatoxigenic *A*. *flavus* after 48 h of incubation at 30°C. **(a)** *Loig*. *coryniformis* BCH-4 CFS. **(b)** *Loig coryniformis* BCH-4 CFS after different treatments [A] proteinase K [B] neutralization at pH 7 [C] pepsin [D] heat treatment (at 100°C for 20 min) [E] catalase treatment [F] MRS broth [G] sterile distilled water.

The CFS of *Loig*. *coryniformis* BCH-4 showed 16.33 ± 0.57 mm inhibition zone against *A*. *flavus* compared to negative control (no zone of inhibition) at 30°C after 48 h (Fig 1A). Besides, the CFS was found to be heat resistant because it did not lose its antifungal activity (16.56 ± 1.56 mm zone of inhibition) after heat treatment against *A*. *flavus* growth. However, the antifungal activity was decreased after treatment with proteinase K (10.66 ± 0.47 mm zone of inhibition) and pepsin (3.44 ± 0.56 mm zone of inhibition), suggesting the presence of proteinaceous compounds in CFS. Moreover, no inhibitory effect was observed at neutral pH which indicated that most of antifungal compounds, present in the CFS of *Loig*. *coryniformis* BCH-4, were pH dependant. Similarly, no inhibitory zone was observed after treatment with catalase. The results of all treatments were compared with negative control (MRS broth and distilled water), as shown in Fig 1B.

### Bioprotection of maize grains by culture supernatant of *Loig*. *coryniformis* BCH-4

**Fig 2:**
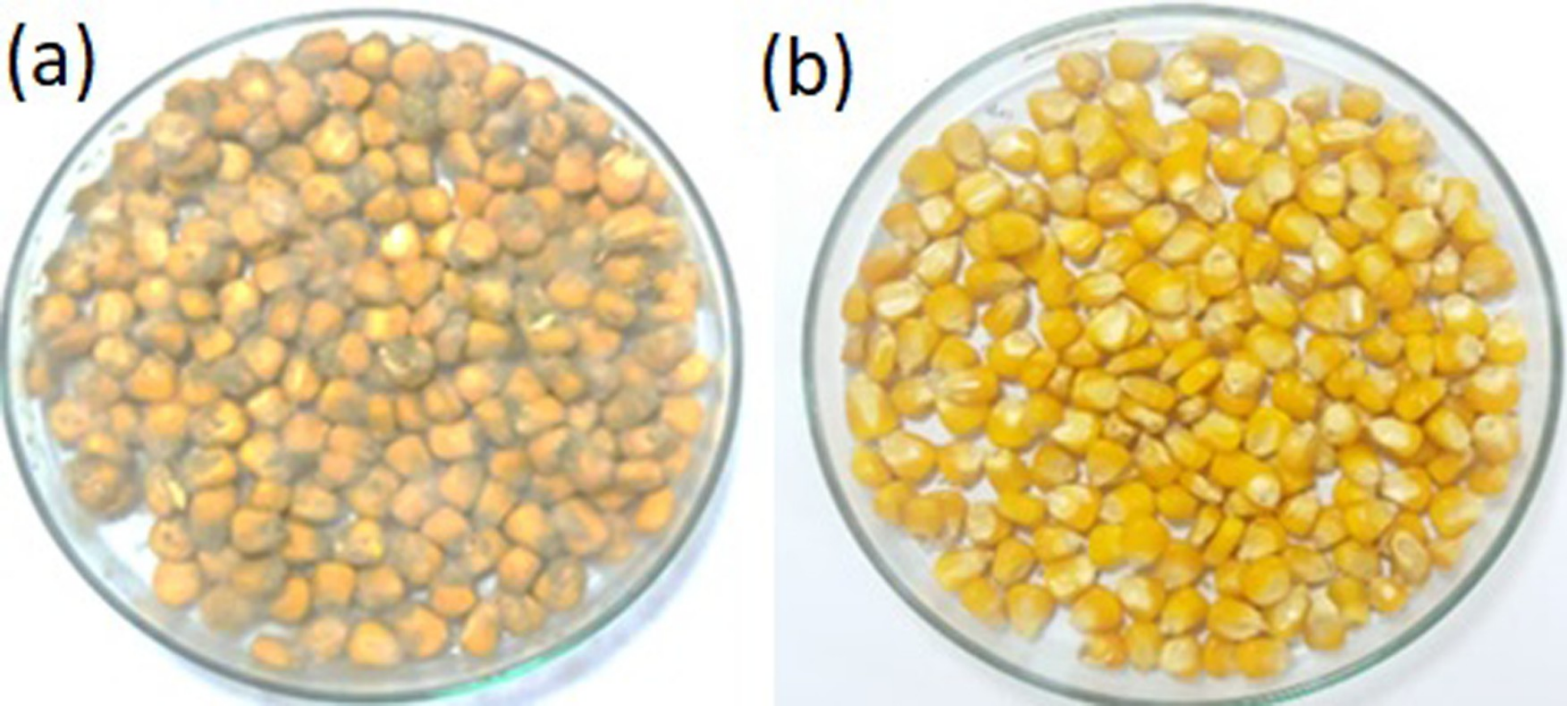
*Aspergillus flavus* growth on to maize grains. (a) treated with MRS broth (control) and (b) treated with CFS of *Loig*. *coryniformis* BCH-4 incubated at 30°C.

The *Loig*. *coryniformis* BCH-4 cell-free supernatant (CFS) showed potent bioprotective effect against aflatoxigenic fungus. Green spores of *A*. *flavus* appeared on MRS broth treated maize grains, showing clear fungal spoilage (Fig 2A). However, no fungal growth was observed on CFS-treated maize grains after seven days of incubation (Fig 2B), thereby confirming the bioprotective potential of *Loig*. *coryniformis* BCH-4.

### FT-IR analysis of maize grains

**Fig 3:**
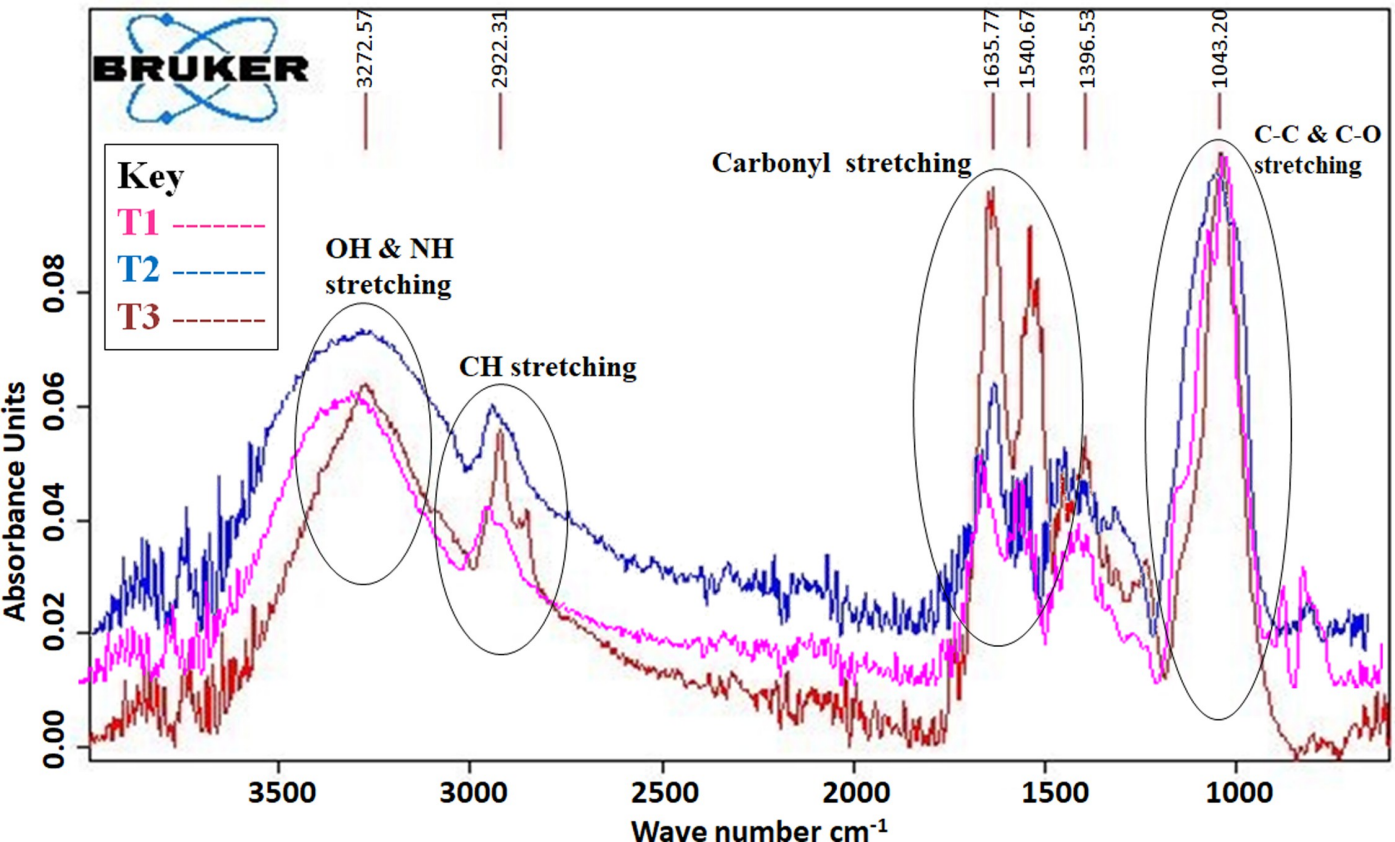
FT-IR spectra of the T1 (untreated maize grains; Pink line), T2 (MRS broth + *A*. *flavus* treated maize grains; Blue line), and T3 (CFS + *A*. *flavus* treated maize grains; Brown line).

The FT-IR spectra of T1 (untreated maize grains), T2 (MRS broth + *A*. *flavus* treated maize grains), and T3 (CFS + *A*. *flavus* treated maize grains) were interpreted within the spectral region of 4000–400 cm^−1^. The absorption bands that appeared in the range of 3500–3000 cm^−1^ are assigned to be–OH and NH stretching [42] and the absorption band in the region of 2922 cm^−1^ can be assigned to be the -CH stretching. Absorption bands 1635 cm^−1^ and 1540 cm^−1^ showed the presence of carbonyl stretching (C = O) [35] while absorbance at 1043 cm^-1^ indicated the presence of C-O-C, C-C and C-O functional groups [42, 43]. In the spectra, the–OH, NH, C = O stretching indicated the presence of peptides linkage (protein contents), CH stretching showed the presence of fatty acid contents while C-O-C, C-C and C-O stretching indicated the presence of carbohydrate contents. The spectra of T1, T2, and T3 maize grains showed similar bands but the patterns and intensities were found to be different (Fig 3). They indicated that nutritional contents (proteins, lipids, and carbohydrates) were decreased in T2 maize grains and affected by the *A*. *flavus* infestation. But in T3, the absorbance of maize grains was increased compared to T1 and T2 grains due to the addition of CFS.

### Total phenolic and flavonoid contents in untreated and treated maize grains

**Fig 4:**
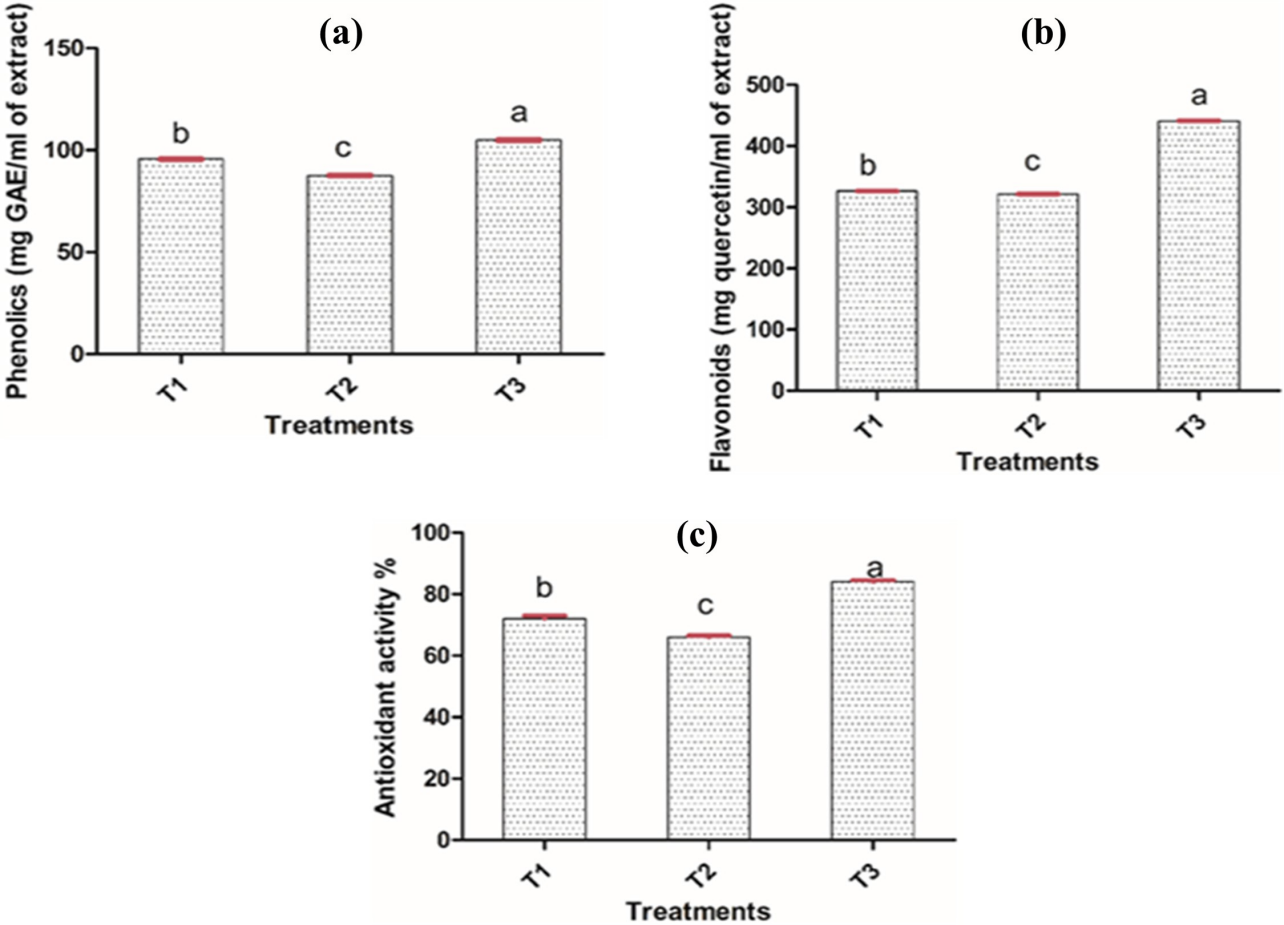
(a) Total phenolics, (b) total flavonoid contents, and (c) total antioxidant activity of T1, T2, and T3 treatment solutions of maize grains. One-way ANOVA (Tukey’s test; p ≤ 0.05) was applied for the analysis of data. Different lowercase letters on bars represent significant differences among treatments (n = 3).

Total phenolic contents were significantly improved in T3 (104.93 mg GAE/mL of extract) compared to T1 (95.63 mg GAE/mL of extract), while decreased total phenolic contents (87.62 mg GAE/mL of extract) were observed in T2 compared to T1 (Fig 4A). It was also noted that the total flavonoid contents were also significantly improved in T3 (441.15 mg quercetin/mL of extract) as compared to T1 (326.28 mg quercetin/mL of extract) whereas, decreased flavonoid contents were observed in T2 (321.48 mg quercetin/mL of extract) compared to T1 (Fig 4B). Moreover, the Tukey’s multiple comparison test was applied to check which treatments means were significantly varying from each other. It was revealed that T1, T2 and T3 were significantly varied (p < 0.05) from each other (S1 Table).

### DPPH scavenging assay of untreated and treated maize grains

Antioxidant activity of treatment solutions (T1, T2 and T3) was evaluated by DPPH scavenging assay. It depicted that T3 have significantly higher (84%) antioxidant contents as compared to T1 (72%). Furthermore, the antioxidant activity of T2 treatment solution was significantly decreased (66%) due to the presence of fungus in comparison with T1 and T3 treatment (Fig 4C). The results of Tukey’s multiple comparison test also indicated the significant difference (p < 0.05) between treatments T1, T2, and T3, because the p-value of T1 vs T2, T1 vs T3 and T2 vs T3 is less than the 0.05 (S1 Table).

### Determination of aflatoxins in untreated and treated maize grains

The aflatoxins (AFB1 and AFB2) were not observed in T1 and T3 maize grains extracts, even after seven days of incubation while in T2 maize grains, the concentrations of AFB1 and AFB2 each were 487 and 16 ng/g respectively (S1 Fig), due to the infestation of aflatoxigenic *A*. *flavus*. A linear curve, in the range of 50–500 ppb of aflatoxins standards solutions, with coefficient of determination (R^2^) of 0.9998, was obtained, using linear regression equation, based on the peak area of each peak, for the analysis of aflatoxins.

### Determination of phenolics / organic acids in CFS of *Loig*. *coryniformis* BCH-4

The HPLC analysis of *Loig*. *coryniformis* BCH-4 CFS revealed the presence of seven phenolic /organic acids (S2 Fig). These resultant phenolic acids cumulatively improved the antioxidant activity of the treatment solution (T3) by improving the antioxidant contents. The phenolics / organic acids profile of *Loig*. *coryniformis* BCH-4 along with their respective retention times and concentrations are presented in Table 1.

**Table 1:** HPLC analysis of phenolics / organic acids in cell free supernatant (CFS) of *Loig*. *coryniformis* BCH-4.

Sr. No.	Phenolic acids	Molecular weight	Retention time	Concentration
		(g/mol)	(min)	(μg/g)
**1**	Chlorogenic acid	354.31	2.703	235.92
**2**	*p*-Coumaric acid	164.16	3.207	114.02
**3**	4-Hydroxybenzoic acid (HB acid)	138.12	6.407	48.36
**4**	Caffeic acid	180.16	7.508	25.55
**5**	Sinapic acid	224.21	12.312	16.99
**6**	Salicylic acid	138.12	15.302	28.70
**7**	Benzoic acid	122.12	18.269	4.14

## Discussion

The bioinfestation of plants and cereals is an important issue. Alternate methods for bioprotection of plants and cereals are being studied for sustainable agriculture worldwide. Many synthetic chemicals have been evaluated and used. However studies have shown health hazards and environmental issues regarding the use of such chemicals [44]. The bioactivity studies of probiotic microorganisms for applications, as natural preservative for food products and their eco-friendly effect on human health, remain to be of great interest [45].

The present study highlighted the bioactivity of *Loig*. *coryniformis* BCH-4 CFS by observing its antifungal potential against aflatoxigenic *A*. *flavus* strain, under different treatments (Fig 1B), in comparison with untreated CFS against the toxigenic *A*. *flavus* strain, shown in Fig 1A. The culture free supernatant of *Loig*. *coryniformis* BCH-4 exerted growth resistance of *A*. *flavus* (Fig 1A). These results have been supported by similar findings for other species of lactic acid bacteria such as, *L*. *plantarum* BCH-1 [29], *L*. *plantarum* RS2 [46], and *L*. *kefiri* FR7 *etc*. [47].

The current results showed no loss in activity after heat treatment at 100°C for 20 min which suggested that the antifungal compounds, present in CFS, are heat resistant. Similar findings have also been reported by Nayyeri et al. [48] that heat treatment did not influence the antifungal activity of CFS of *L*. *plantarum*. However, the activity of *Loig*. *coryniformis* BCH-4 CFS decreased against *A*. *flavus* after treatment with proteinase K and pepsin, suggesting the presence of proteinaceous antifungal compounds present in the CFS of *Loig*. *coryniformis* BCH-4 [33]. Comparably, after neutralizing the pH of CFS, no zone of inhibition was observed against *A*. *flavus*. These results are consistent with Wang et al. [49] who studied anti-fungal properties of *Lactobacillus* isolates from kumis against *Penicillium roqueforti* and *A*. *niger*. Their findings showed that an increase in pH (from 3.8 up to 7) ultimately reduced the size of inhibition zone. Therefore, antifungal activity of LAB is based on several mechanisms such as organic acids production and hence pH variation or pH-dependent antifungal proteins [33]. Likewise, during the treatment of CFS with catalase, no inhibitory effect was evaluated against *A*. *flavus*; this verified the involvement of hydrogen peroxide as antifungal compound which was converted into oxygen and water by this enzyme [33].

Based on the finding of antifungal activity, CFS of *Loig*. *coryniformis* BCH-4 was employed for bioprotection of post-harvested maize grains, infected with *A*. *flavus* (Fig 2). The bioprotection effect in the current study could be attributed to various bioactive compounds, present in *Loig*. *coryniformis* BCH-4, namely organic acids, fatty acids, phenolic compounds, proteinaceous compounds *etc*. mainly known for their antifungal potential [21, 50, 51]. In our previous work, the organic acids; pyruvic acid, lactic acid, citric acid, malic acid, succinic acid, and malonic acid have been reported in *Loig*. *coryniformis* BCH-4, for their potent antifungal effects against *A*. *flavus* and *A*. *fumigatus* [31]. Other *Lactobacillus* species such as *L*. *casei*, *L*. *rhamnosus*, *L*. *plantarum*, *L*. *paracasei* and *L*. *curvatus* also produced lactic acid, acetic acids, formic, citric, succinic and glutamic acids [52]. Other kind of compounds namely, cyclic dipeptides *cis*-cyclo (L-Val-L-Pro), *cis*-cyclo (L-Phe-L-Pro), cyclo(Leu-Leu) and cyclo(L-Leu-L-Pro), produced by *L*. *plantarum* and *Loig*. *coryniformis*, have also been reported for antifungal activity against *Ganoderma boninense* and *A*. *flavus* [25, 53, 54]. We have also reported previously 12-hydroxydodecanoic acid, a fatty acid, having antifungal activity against *A*. *flavus* and *A*. *fumigatus* in Bukhari et al. [29]. Other four hydroxy fatty acids; 3-hydroxydecanoic acid, 3-hydroxy-5-cis-dodecanoic acid, 3-hydroxydodecanoic acid, and hydroxytetradecanoic acid from *L*. *plantarum* milab14 have also been reported for their antifungal activities [55]. The fungal inhibitory mechanism of *Loig*. *coryniformis* BCH-4 CFS includes membrane destabilization, proton gradient interference, enzyme inhibition, and creation of reactive oxygen species [26, 56].

The FT-IR analysis (Fig 3) indicated the effect of *A*. *flavus* on basic macronutrients of maize grains like proteins, fats and carbohydrates, by observing their relative intensities of characteristic absorption peaks. The decreased intensities in T2 treatment of subject nutrients might be due to fungal breakdown of such nutritional components into smaller fragments that were further metabolized [57]. The complex protein molecules are also utilized for obtaining energy, by respiration and to synthesize hyphal wall in fungi [11, 58]. In T3 treatment, the CFS of *Loig*. *coryniformis* improved the nutritional contents of maize grains and protected them from fungal attack, presumably due to the addition of CFS, that is a mixture of phenolic compounds, fatty acids, organic acids, and peptides *etc*. [59, 60].

More precisely, the culture supernatant of *Loig*. *coryniformis* BCH-4 not only guarded against *A*. *flavus* infestation but also augmented with improved antioxidant contents of T3 treated maize grains (T3: CFS + *A*. *flavus*). The results revealed a significant improvement (p ≤ 0.05) in total phenolic contents (104.93 mg GAE/mL of extract), total flavonoid contents (441.15 mg quercetin/mL of extract), and total antioxidant activity (84%) (Fig 4) of T3 treatment in comparison with T1 (untreated) and T2 (MRS broth + *A*. *flavus* treated). Similar findings of bioprotection have previously been reported for *L*. *plantarum* in soybeans and maize grains, against *A*. *flavus* [25, 34, 40]. Despite this, the present study first time described the significant improvement of antifungal potential in CFS treated T3 maize grains by improving its functional antioxidant contents. This finding was supported by our previous study [31] that the CFS of *Loig*. *coryniformis* BCH-4 had potent antioxidant potential by observing its DPPH scavenging activity. Moreover, other species of *Lactobacillus* (*L*. *plantarum*, *L*. *helveticus*) also had reported potent antioxidant activity [61]. The increased antioxidant contents, in T3 maize grains, was justified due to the addition of *Loig*. *coryniformis* BCH-4 CFS, a mixture of metabolites, including phenolic compounds [59, 62]. Besides, in T2 treatment solution, the antioxidant contents (phenolics and flavonoids) and antioxidant activity were significantly (p ≤ 0.05) decreased as compared to T1 and T3. It is believed that the observed decrease was due to the action of fungal polyphenol oxidases that catalyzes the oxidation of many antioxidant compounds including phenolics and flavonoids into quinones [63]. The process of oxidation badly affects the food components including degradation of many bioactive food compounds such as phenolics and flavonoids, along with loss of its nutritional values [14].

Moreover, aflatoxins analysis revealed a complete inhibitory production of AFB1 and AFB2 in T3 treatment, due to the presence of various bioactive compounds, present in the CFS of *Loig*. *coryniformis* BCH-4, in comparison with T2, having aflatoxins (AFB1 and AFB2) level of 487 and 16 ng/g respectively (S1 Fig). The results suggested that metabolic products of *Loig*. *coryniformis* BCH-4, based on their ability to decrease the growth of aflatoxigenic *A*. *flavus* and biosynthesis of aflatoxins, could be considered as a good agent for bioprotection of maize grains. Previously Nazareth et al. [34] has reported that *L*. *plantarum* CECT 749 CFS reduced the AFB1 level (6.9 ng/g) in maize as compared to the control group (278.4 ng/g). Moreover, some other previous studies have also shown the potential inhibition of *A*. *flavus* aflatoxins (AFB1) production by *L*. *casei* and *L*. *plantarum* in maize [34, 40, 64] while the current study describes the inhibition of *A*. *flavus* aflatoxins, using *Loig*. *coryniformis* BCH-4 CFS. The current finding was also supported by several other studies like *L*. *plantarum* AF1 that inhibit the growth and aflatoxins production of *A*. *flavus* in soybeans [25]. In another study, Kachouri et al. [65] found *L*. *plantarum* to be effective against *A*. *flavus* in olives by reducing AFB1 levels.

The HPLC analysis of *Loig*. *coryniformis* BCH-4 CFS showed the presence of chlorogenic acid, p-coumeric acid, 4-hydroxybenzoic acid (HB acid), caffeic acid, sinapic acid, salicylic acid, and benzoic acid (S2 Fig). The presence of these phenolic acids in CFS ultimately revealed an increase in total phenolic contents and antioxidant activity of T3, which can be directly augmented with bioprotection of maize grains. Nazareth et al. [34] identified phenolic acids such as Gallic acid, protocatechuic, chlorogenic acid, vanillin, sinapic acid, salicylic acid, and p-coumaric acid in *L*. *plantarum* CFS and documented for potent antifungal activity against *A*. *flavus* and *Fusarium verticillioides*. These identified phenolic acids have previously been reported as antifungal compounds [34].

The chlorogenic acid and p-coumaric acid were present in high concentrations, which have been previously reported for disrupting the fungal cell membrane structure [66, 67]. Overall, the presence of phenolics / organic acids in CFS of *Loig*. *coryniformis*, improved the nutritional contents of maize grains and also contributed to increase the antifungal potential along with other bioactive metabolites such as organic acids, proteins, peptides, fatty acids etc., that are extensively considered as antifungal compounds [20, 27].

## Conclusions

The present work explored the antiaflatoxigenic potential of *Loig*. *coryniformis* BCH-4. The results highlighted no production of aflatoxins in CFS treated maize grains even after seven days of infestation with *A*. *flavus*. Besides, this treatment also improved the antioxidant contents and antioxidant activity of maize grains which might be due to the presence of various phenolic acids (chlorogenic acid, *p*-coumaric acid, 4-hydroxybenzoic acid, caffeic acid, sinapic acid, salicylic acid, and benzoic acid) in the CFS of *Loig*. *coryniformis* BCH-4. To the best of our knowledge these phenolic acids have not been reported earlier in this bacterium, therefore, provide conclusive evidence as biopreservative, by enhancing nutritional and functional antioxidant values of *Zea Mays L*. The results also provide new insights for the biotechnological employment of such probiotic bacteria as bioprotectant for other cereal grains in future.

## Supporting information

S1 Table Tukey’s multiple comparison test between treatments (T1, T2 and T3). (DOCX)

S1 Fig Detection of aflatoxins in T2 treatment of *Zea mays* L. grains extract. During HPLC analysis, the concentrations of detected aflatoxins AFB1 and AFB2 were 487 and 16 ng/g respectively, in T2 treatment (b). However, the aflatoxins were not detected in T1 and T3 (a) and (c).
(DOCX)

S2 Fig HPLC analysis of phenolics / organic acids. (a) Phenolic / organic acids in CFS of *Loig*. *coryniformis* BCH-4. (b) Standards of phenolics / organic acids.
(DOCX)
