# Supplementary material for: Bioprotection of Zea mays L. from aflatoxigenic Aspergillus flavus by Loigolactobacillus coryniformis BCH-4
Source: PLoS One. 2022 Aug 2;17(8):e0271269. doi: 10.1371/journal.pone.0271269 (PMC9345345; doi:10.1371/journal.pone.0271269)
Supplement: S1 Table — (DOCX) [file pone.0271269.s001.docx]

# Supporting Information

**S1 Table.** **Tukey's Multiple Comparison Test between treatments (T1, T2 and T3)**

| Total phenolic contents | | | |
| --- | --- | --- | --- |
| Tukey's Multiple Comparison Test | **Mean Difference** | **Significant; P-value < 0.05** | **95 % CI** |
| T1 vs T2 | 8.010 | Yes | 6.232 to 9.788 |
| T1 vs T3 | -9.297 | Yes | -11.07 to -7.519 |
| T2 vs T3 | -17.310 | Yes | -19.08 to -15.53 |
| Total flavonoid contents | | | |
| Tukey's Multiple Comparison Test | **Mean Difference** | **Significant; P-value < 0.05** | **95 % CI** |
| T1 vs T2 | 4.793 | Yes | 2.838 to 6.749 |
| T1 vs T3 | -114.870 | Yes | -116.8 to -112.9 |
| T2 vs T3 | -119.663 | Yes | -121.6 to -117.7 |
| Total antioxidant activity | | | |
| Tukey's Multiple Comparison Test | **Mean Difference** | **Significant; P-value < 0.05** | **95 % CI** |
| T1 vs T2 | 6.010 | Yes | 2.762 to 9.258 |
| T1 vs T3 | -11.990 | Yes | -15.24 to -8.742 |
| T2 vs T3 | -18.000 | Yes | -21.25 to -14.75 |
