# Supplementary material for: Bioprotection of Zea mays L. from aflatoxigenic Aspergillus flavus by Loigolactobacillus coryniformis BCH-4
Source: PLoS One. 2022 Aug 2;17(8):e0271269. doi: 10.1371/journal.pone.0271269 (PMC9345345; doi:10.1371/journal.pone.0271269)
Supplement: S1 Fig — During HPLC analysis, the concentrations of detected aflatoxins AFB1 and AFB2 were 487 and 16 ng/g respectively, in T2 treatment (b). However, the aflatoxins were not detected in T1 and T3 (a) and (c). (DOCX) [file pone.0271269.s002.docx]

# Supporting Information

**S1 Fig.**


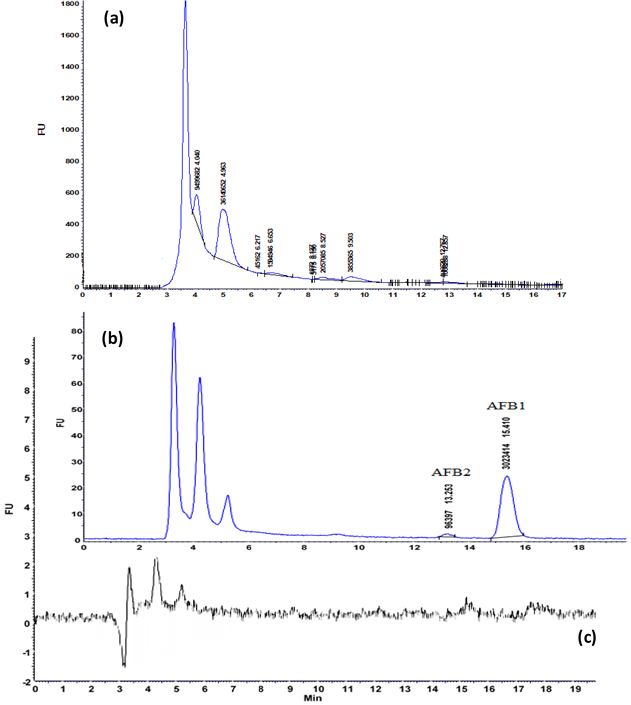


**S1 Fig: Detection of aflatoxins in T2 treatment of *Zea mays* *L* grains extract.**

During HPLC analysis, the concentrations of detected aflatoxins AFB1 and AFB2 were 487 and 16 ng/g respectively, in T2 treatment (b). However, the aflatoxins were not detected in T1 and T3 (a) and (c).
