# Supplementary material for: Bioprotection of Zea mays L. from aflatoxigenic Aspergillus flavus by Loigolactobacillus coryniformis BCH-4
Source: PLoS One. 2022 Aug 2;17(8):e0271269. doi: 10.1371/journal.pone.0271269 (PMC9345345; doi:10.1371/journal.pone.0271269)
Supplement: S2 Fig — (a) Phenolic / organic acids in CFS of Loig. coryniformis BCH-4. (b) Standards of phenolics / organic acids. (DOCX) [file pone.0271269.s003.docx]

# Supporting Information

**S2 Fig.**


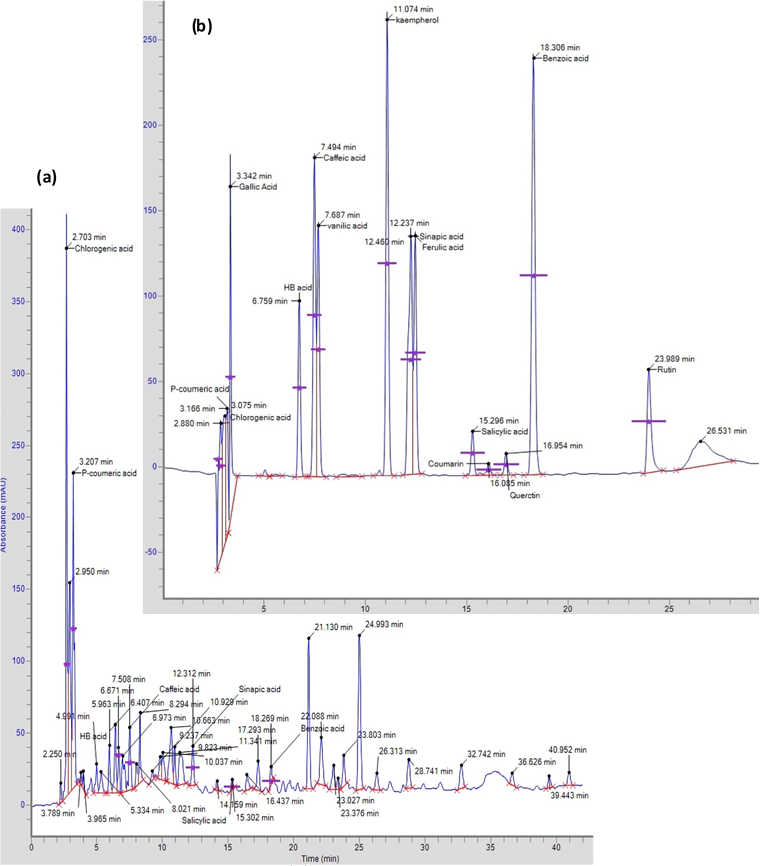


**S2 Fig: HPLC analysis of phenolics / organic acids** (a) Phenolic / organic acids in CFS of *Loig. coryniformis* BCH-4. (b) Standards of phenolics / organic acids.

The HPCL chromatogram depicts time on X-axis while absorbance on Y-axis. Various phenolics *i.e.,* Chlorogenic acid, p-coumaric acid, 4-hydroxybenzoic acid, Caffeic acid, Sinapic acid, Salicylic acid, and Benzoic acid were detected at 2.703, 3.207, 6.407, 7.508, 12.312, 15.302, and 18.269 min respectively.
